# Improving Medical Tourism Services through Human Behaviour and Cultural Competence

**Published:** 2019-11

**Authors:** Ladan ROKNI, Sam-Hun PARK, Turgay AVCI

**Affiliations:** 1. Asia Contents Institute, Konkuk University, Seoul, South Korea; 2. Faculty of Tourism Management, Eastern Mediterranean University, Famagusta, North Cyprus

**Keywords:** Medical tourism, Cultural competence, Human behaviour, South Korea

## Abstract

**Background:**

Medical tourism is a type of service sector in which there is direct interaction between healthcare practitioners and patient-customers, leading to several challenges due to cultural and social background differences. We aimed to investigate the determinants of delivering a culturally-oriented service in medical tourism sector.

**Methods:**

Adopting an exploratory qualitative approach, interviews were conducted through a semi-structured procedure with authorities across various medical sectors in South Korea in winter 2017. Participants were all involved in and aware of the medical tourism sector, both academically and clinically. The interview transcripts were coded through a systematic thematic analysis.

**Results:**

In order to focus on non-clinical service in medical tourism sector, and a system of cultural competence delivery, three main themes were identified: 1) The personal characteristics of doctors; 2) External supports to be provided by the associated organisations; and finally, 3) Skilfulness, which implies the culturally-oriented interaction with foreign patients.

**Conclusion:**

Several strategies are suggested to address the non-clinical challenges and conflicts in doctor-patient interaction in the sector of medical tourism. It is likely that providing a culturally-oriented service in this sector demands for a comprehensive planning, and several strategies for implementation in order to support and train a team of skilful doctors with non-clinical characteristics. These finding will likely have insights for those organisations searching to improve their performance in the medical tourism sector.

## Introduction

Medical tourism is a new trend in healthcare mobility (1–3) in which people (patient-customers) travel across borders to seek medical treatment or healing. They have different cultural and social background from their care providers. Medical tourists prefer to travel to destinations with comparable culture with their own background, which proves that the cultural factor can play a role as logic for mobility (1, 4–6). In fact, ‘culture’ have been always considered as an efficient determinant in shaping the patterns of medical tourism sector (4), and this sector is named as the most ‘costumer-oriented sector’ in healthcare (1). Providing a ‘culturally-congruent’ service to patient-customers would lead to an appropriate and effective interaction between patient and doctors, which potentially would be followed by satisfaction (7), trust (8), and increased healthcare quality (7). Cultural background is not the only determinant; family and personal preferences, for example, are also likely to impact this interaction. In the healthcare sector, the ability of healthcare providers to deliver culturally congruent services is dependent to the ‘cultural competence’ (4, 9)' of the healthcare workforce (10). A lack of this ability could have adverse consequences including the risk of misdiagnosis arising from miscommunication between the health practitioner and the patient. In order to provide a competitive advantage in the contemporary situation of medical tourism sector, the work of ‘doctoring’ need to be transformed to be ‘physician-healers’ (11). The cultural-oriented interaction will assist the doctors to discover a new perspective to their work, while they are helping patients through the strength of their humanity (12).

Acknowledging that medical tourism is a unique type of service and is highly affected by cultural factors (5, 13), and ‘culture’ plays a pivotal role in designing the behaviour in the healthcare domain (1, 14), it seems that studding the associated aspects of a culturally-oriented care would be efficient in designing a competitive advantage. It will improve the quality of service through reducing tension of foreign patients who are highly vulnerable due to their health situation (15) and have to face added pressure to tolerate cultural differences (16). ‘Cultural competence’ relies on information and ‘knowledge’ of their providers, which is transformed into ‘skills, service approaches, policies, and marketing’ (17). Meanwhile, the dimensions introduced to define and evaluate ‘cultural competence’ suffer from lack of a uniform procedure in the healthcare sector, and there is no consensus on how to deliver and implement cultural competence (4). Therefore, there is a demand to investigate the process of providing culturally-oriented service in the medical tourism sector and its associated factors.

Accordingly, we aimed to study the importance of human behaviour (cultural-oriented factors) in medical tourism sector, and to investigate the associated factors of delivering cultural competence, from healthcare practitioners’ perspectives. This study could potentially lead to a deeper understanding of non-clinical factors in medical tourism and assist the involved organisations in improving their service through focusing on the non-clinical abilities of their healthcare human resources.

## Methods

### Case Study: South Korea

South Korea is well-known as ‘the ultimate destination for smart care’ in medical tourism sector. The image of medical tourism in this country has been built based on the ‘advanced health facilities’, ‘tech-based treatment’, and beauty surgeries (18). Despite designing an accurate system of medical tourism in Korea, this sector has lately failed to return the amount of money invested. What is clear is that Korea insists on a world-class treatment, and the greatest proportion of effort and investment have been given to the development and marketing of the Tech-based medicine. Yet, the brand of Korean medical tourism has been criticized since the advanced technology might not be of interests for all the potential patients-customers (19).

### Research Design and Sample Selection

This exploratory study adopted a qualitative and constructivist approach. A preliminary content analysis was applied to identify the key factors through an extensive review of literature, including cultural competence in medical tourism (15), in healthcare (9, 20), developed models (21, 22), and variations of the components (4).

Provided that healthcare authorities design the policies of medical tourism, we identified this community as the participants through a ‘purposive sampling’. Twelve experts were interviewed in winter 2017 to obtain insight into their experiences with cultural barriers. The inclusion criteria was being involved in a job position which requires direct interaction with foreign patients, and to have some degree of involvement in developing and promoting the quality of medical tourism service in South Korea in their professional positions. A constructivist approach was adopted and the data collection was completed when ‘data saturation’ appeared. To avoid bias, all participants were assured that their personal information would be kept ‘confidential’ and the interviewer tried to be ‘judgmental’ (23).

### Ethical approval

Official authorization was taken from the respective hospitals or organizations.

### Data Analysis

‘Thematic analysis’ was applied to analyse the interview transcripts, following the procedure designed by Luborsky (24). A systematic ‘coding’ was adopted to arrange the data (25), and the themes were emerged based on ‘overarching concepts’ in interviews. Table 1 shows an example of the coding process.

**Table 1:** Qualitative Coding Process, Identification of Themes

***Text***	***Open Codes (examples)***	***Categories***	***Theme***
The relevant notions of the participants mentioned in the text	Familiarity with the verbal and non-verbal expression	Knowledge	Internal characteristics
	Attitude	Attitude	
	Motivation to work	Motivation and desire	
	Commitment to work	Commitment	
	Demand for training	Training	External support
	Demand for support	Organization	
	The ability to follow the regulation and fulfil the needs	Skillfulness	Skill

To ensure trustworthiness, participant feedback and triangulation were used. It involved comparing the results with secondary documents, and the three final participants were asked to confirm or comment on the relevance and clarity of the developed framework.

## Results

While there was a general consensus that ‘training’ and ‘supports’ are required for the health-related human resources, there remained a general agreement that ‘personal efforts’ are also a crucial factor in improving the non-clinical care to patient-tourists. Three main concepts were identified presented in Table 1 as ‘theme’ and are discussed as follows.

### Personal characteristics

To deliver culturally-congruent services to foreign patient-tourists, almost all respondents noted the importance of the personal characteristics of healthcare human resources. These characteristics range from doctors’ ‘awareness’ of cultural differences, ‘familiarity’ with the importance of these issues, ‘desire’ to enhance their abilities, and, not surprisingly, their ‘commitment’ to follow non-clinical regulation, apart from their clinical proficiency.

In the medical tourism sector, the ‘*doctor’s critical characteristics*’ that emerged from the interviews are categorized into knowledge, attitude, motivation and desire, commitment, and hard work.

Many participants agreed that providers’ awareness of the ‘cultural differences’ and ‘personal differences’ of their patients is the most important characteristic. They noted that: ‘*The critical responsibility of doctors* [working with patients-tourist] *is to be familiar with the cultural expression of their patients*’, and also other participants mentioned: ‘*when we have a foreign patient traveling for their treatment, we better know their verbal and non-verbal expression*’. Accordingly, cultural preparation can be helpful in the delivery of cultural competency; a hospital representative also mentioned that ‘*we should get familiar with the differences to be ready to react appropriately in different situations*’. Moreover, it was agreed that the ability of cultural competency is an ‘ongoing process’ and doctors’ awareness of this issue is critical; one participant described healthcare cultural competence as *‘…an on-going process and the doctors should intend to assess the cultural and personal needs of their foreign patients’*. However, other participants discussed that although familiarity with other cultures is crucial; it is not the only determinant since ‘*medical tourism is a novel arena that demands positive feeling from the providers’*. Even though healthcare providers are required to be committed to non-clinical regulation, it is something internal and they cannot be forced to do so. For instance, it was mentioned that ‘*in some cases the hospitals aim to provide a cultural-based service, but the doctors are not willing to follow the path although they are familiar with the requirements*’. Another participant noted that *‘We can train individuals to treat foreign patients, but they should potentially be willing and motivated to do so’*.

The majority of interviewees discussed the importance of having a positive attitude toward foreigners, constructive motivation, and willingness to continue and improve the quality of interaction. Accordingly, the two determinant characteristics that emerged in the interaction between doctors and patient-tourists were ‘positive attitude’ and ‘motivation’, as it was indicated that doctors, are required to be open-minded and aware of the cultural importance through ‘*respecting the beliefs of patients from different cultures*’, and also ‘*respecting different races*’, another participant described it as ‘*being positive about the importance of cultural oriented services*’.

When it comes to non-clinical ‘motivation’, it was agreed that those doctors who are ‘*willing to interact with patients from culturally diverse groups*’ and who are ‘*seeking cultural training*’ will deliver a culturally-oriented service with better quality. Also, it stems from the fact that they are ‘*passionate to learn*’ and willing to ‘*enhance their abilities*’.

Another critical characteristic discussed was the ability to be ‘*hardworking and committed*’ which is an essential characteristic and results from the fact that commitment to offering a culturally-oriented service will lead to higher satisfaction of patients. It was noted that ‘*interpreters help doctors and patients, but in those cases that doctors also try to be involved in, patients are more satisfied’*. Hence, it was believed that it is necessary for doctors to *‘continuously improve their cultural abilities’.*

### External Supports

All participants made it clear that providing a culturally-oriented healthcare service in the medical tourism domain is at its ‘*early stages*’, specifically in South Korea. They agreed that it is ‘*complicated*’ to present a culturally congruent service to patients who are tourists as well, and then ‘training’ plays a crucial role to assist doctors; as one physician noted: ‘*there is lack of a clear path for* [healthcare] *providers to follow*’. It is also not limited to university education, since one participant mentioned: ‘*Although the basic lectures in university inculcate a sense of cultural competence, still we need to design a well-established system to direct doctors in this fast changing system*’.

Even though there was a general acceptance that cultural training is essential, it would be impossible to generalize a specific training to all members of a society, and the creativity will be a role in order to recall the training contents and at the same time to employ their ‘personal characteristics’. It was mentioned that ‘*we cannot generalise a characteristic to all societies or cultures*’ and one participant noted:

*‘Cultural competency cannot be established as a specific package of standards; rather, the doctors can be assisted or guided by a designed education system and supports’.*

Educational assistance should be provided by relevant committees, ranging from governments to private providers, and also through ‘*a comprehensive policy*’. It was agreed that ‘*there is a need to help them* [doctors] *to keep their standards* [non-clinical and cultural service] *up to date*’. This support might assist doctors ‘*to get familiar with non-verbal expression for integrating with overseas patients’*. Perceived organisational support might happen by ‘*encouraging doctors to improve their cultural competency*’ and also by developing ‘*an environment in hospitals with the trend of cultural competence*’. It was believed that training and support are not only required at the individual level, but there is also a demand in hospitals that are involved in medical tourism. As one participant mentioned:

*‘Hospitals are still not convinced why they should spend money for education... the government is providing free cultural training sessions with the aim of improving the skills of employees’.*

### Skill

A successful delivery of cultural competence from doctors to patient-tourists could not be guaranteed without the essential role that a ‘*skilful*’ health human capital can play; ‘*even the training cannot be effective without being wise enough to know how to respond at the time of interaction’*. When it comes to cultural interactions in medical tourism sector, skilful doctors need to be able to apply not only the previously learned non-clinical techniques, but also be aware enough of the importance of their characteristics at the same time. The participants agreed that ‘*it is important* *to learn, evaluate, and analyse the situation*…*for each foreign patient-tourist individually*’ who is coming from a different cultural background and has specific individual needs.

**Fig. 1:**
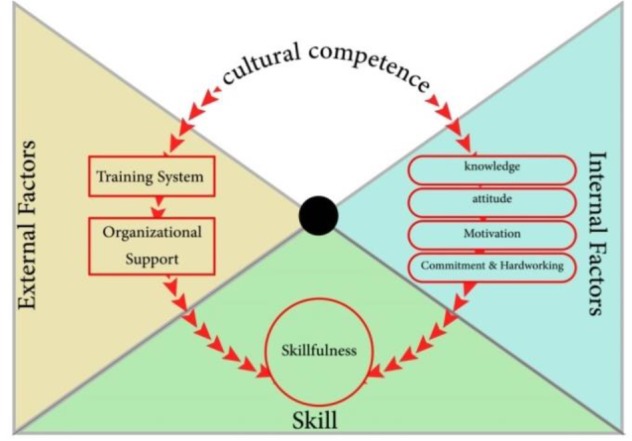
A Framework of Delivering Cultural Competence in Medical Tourism

Figure 1 is a framework designed based on the theme analysis, representing the determinants of cultural competence in the scope of medical tourism.

## Discussion

This study focused on the influence of human behaviour in medical tourism sector, and specially to investigate those factors with a potential of contributing to delivering culturally-oriented service, with a focus on the interaction between Korean physicians and the patient-tourists.

The sector of medical tourism is the most ‘customer-oriented’ sector in healthcare, and cultural factors besides the geographical factors can impact the rationales of mobility (1). Cultural familiarity is considered as a determinant in destination decision making process (26) and the ‘patterns’ of medical tourism (4). The ‘lens of culture’ and ‘human behaviour’ (27) play a pivotal role in the perceived definitions in both healthcare and tourism sector, respectively, and it also can improve the quality of service (28, 29), and eliminate racial/ethnic disparities’ (12). Moreover, it is believed that healthcare sector is ‘commodified’ (1) and medical tourism is far from a ‘uniform’ developmental model (2). Therefore, in the current competitive situation, focusing on the non-clinical factors can play a pivotal role in the destinations ability to capture the right market and will assist both organisations and the human capital.

Consistent with the previously developed model in healthcare domain (30, 31), the participants tended toward a view of cultural competence in which the abilities of providers can be determined by their personal characteristics and through external assistance. The most critical individual characteristics for delivering a culturally congruent service are: the ‘knowledge’ of considering cultural competence as an ‘ongoing process’ (9, 32) which takes time and effort (30). Moreover, due to the ‘vulnerable’ situation (15) that every single patient-tourist and their family are faced with and the ‘added pressure’ (16) due to the differences, it is likely that the ‘medical’ proficiencies of doctors are insufficient to address the non-clinical needs of patient-tourists and ‘physician-healers’ can fulfil the patients’ needs in a better quality (11). Indeed, their lack of ‘familiarity’ with other cultures (33) and failing to present a positive ‘attitude’ (9, 29) toward cultural and personal differences will impact satisfaction (7, 34) and treatment quality (28, 29). The other personal characteristics, mentioned by the participants, were ‘motivation (21) and ‘desire’ (9, 30). These characteristics refer to those doctors who are passionate about interacting with patient-tourists and are seeking the training to improve their non-clinical experience. Finally, it is likely that working in the medical tourism sector demands a team of doctors who are ‘committed’ to the newly established non-clinical regulations by their organisations and are ‘hardworking’ enough to invest their time and effort into improving their non-biomedical abilities.

Health human resources involved in medical tourism sector are assets who can ease the process of cultural competence delivery through their individual characteristics. Yet, there are difficulties to convince doctors about the importance of the cultural factors during the treatment since it might be seen as a soft social science type thing or not as important as medicine (12). Accordingly, external supports were introduced which is combined of the ‘cultural training’ and the necessity of ‘organizational support’. Organisational support was previously introduced as a main dimension of cultural competence evaluation in healthcare (30) that impacts the cultural competence behavioural scores (35). Likewise, cultural competence was introduced as an organisational strategy and innovative policy (36), which seems logical enough to consider it as a remedy in the competitive situation. Even though there is a dearth of evidence in healthcare literature on the association of cultural-diversity training and healthcare quality (37), it might positively influence the level of awareness, knowledge, behaviour, and attitude of healthcare providers, as well as their skills (38).

This process could not be guaranteed unless the presence of the skilful doctors at the time, where they try to apply their own knowledge, analyse the situation, and interact accordingly with every each patient. Indeed, skill was one of the key factors of cultural competency (4) in almost all the developed models and implies on the abilities of doctors who intend to locate the cultural needs of their patient-tourists based on their personal abilities and the external assistance. The emergent themes in this study are consistent with those of the developed cultural competence models in healthcare (21, 30). However, there are some variations in the involved factors, which are likely to be caused by the type of treatment (30) or geographic and ethnic background (3).

In Asian countries, the trend is, mostly, going forward to the modern medicine treatment and better quality (39). South Korean healthcare system has been always linked with advances in technology (14). However, the effectiveness of Korean policy in medical tourism marketing and promoting has been lately criticized (19, 40). There are social implications to the new emerged trend since it potentially can impact the importance of the communication, and then the competitiveness of a destination. Patient-costumers are seeking for a culturally congruent service along with a high-quality medicine. The contemporary situation demands for a cultural-based remedy since South Korea tends to apply the Tech-based advances in its medical tourism branding, however, it is believed that ‘human behaviour’ will dictate the future of medical tourism marketing, noted by Geva, 2018 (40). In order to provide the culturally-oriented service, a comprehensive planning is required which is followed by monitoring the implementation. It would not take place unless all human capital associated with medical tourism get involved in a well-established training and promoting approach. The emergent factors are likely to be rational in regard to the current situation in South Korea, as an ‘entrepreneurial’ type of government (1, 18). Governmental support has lately been not only in the form of financial support but also they provide individual and organizational training to increase the awareness (41). The associated organizations supervise the operation of medical tourism through a persuasive policy and the formulation and implementation of these policies.

## Conclusion

In the contemporary situation of medical tourism sector which is highly competitive, there is a lack of an innovative privilege for each destination. This sector involves vulnerable customers who have different cultural and social background and there is high demand for those unwritten regulations that are centralized on doctor-patients interaction. Hence, cultural factors would provide competitive advantages through focusing on non-clinical factors and providing culturally-oriented service. Addressing the cultural needs cannot happen unless there is a team of healthcare human resources who are proficient and possess both clinical and non-clinical abilities. Cultural competence training can be an investment in marketing medical.

The developed framework adds to a growing body of knowledge on the non-clinical factors in the medical tourism sector, especially with regards to healthcare human resources. The similarities suggest that our findings may have particular relevance to organisations looking to grow medical tourism sector. In the contemporary situation of South Korea, it seems logical to invest into healthcare workforce improvement in regard to their non-clinical abilities, and the government can reconfigure the policy, especially in terms of promotional support.

## Ethical considerations

Ethical issues (Including plagiarism, informed consent, misconduct, data fabrication and/or falsification, double publication and/or submission, redundancy, etc.) have been completely observed by the authors.
